# How much behaviour change is required for the investment in cycling infrastructure to be sustainable? A break-even analysis

**DOI:** 10.1371/journal.pone.0284634

**Published:** 2023-04-19

**Authors:** Paolo Candio, Emma Frew

**Affiliations:** 1 Department of Economics and Management, University of Trento, Trento, Italy; 2 Centre for Economics of Obesity, Institute of Applied Health Research, University of Birmingham, Birmingham, United Kingdom; Mississippi State University, UNITED STATES

## Abstract

**Background:**

Active travel has gained traction among policy makers as a promising solution to physical inactivity. Returns on active travel investments, including cycling infrastructure, crucially rely on resulting improvements in population behaviours. Estimating the expected economic value that an additional regular cyclist will generate and being able to identify the behaviour change required at the population level to offset the intervention costs is important to inform future investment decisions.

**Methods:**

The WHO’s Health Economic Assessment Tool was employed to conduct a break-even analysis. A case study methodology was used which focused on a real-world construction project of a separated cycleway in the UK. The economic assessment considered physical activity benefits, air pollution, crash risk and carbon emissions in monetary terms. An iterative computational approach was applied to identify the behaviour change (cycling) requirements, and corresponding benefits valued using international dollars, to break even on the investment costs. Sensitivity analyses were conducted to assess robustness of the base-case results.

**Results:**

Over a ten-year time horizon, an additional regular cyclist (i.e., someone cycling most days of the week) was found to generate $798 (₤533) per annum (international dollars). An additional 267 regular cyclists per km were required to break even on the construction of the new separated cycleway. Estimates were particularly sensitive to variations to age, cycling volume and evaluation time horizon.

**Conclusions:**

Policymakers planning to invest in cycling infrastructure should consider using these reproducible, order-of-magnitude estimates to complement the more comprehensive transport appraisal and budget allocation processes. This would ensure that, when considering its health-related economic benefits, the investment is justifiable on economic sustainability grounds.

## Introduction

Physical inactivity is a major public health issue for many countries around the world [1]. Large sections of the population are insufficiently active [2], putting undue pressure on national health systems and budgets [3]. Physical inactivity is a leading risk factor for noncommunicable diseases and mortality [4]. It increases the risk of several chronic health conditions, including type II diabetes [5], cardiovascular disease [6], colorectal cancer [7] and depression [8]. The global cost of physical inactivity to healthcare systems alone has been conservatively estimated at $53.8 (international dollars) billion in 2013 [9], with the negative health consequences being borne disproportionately by more disadvantaged groups [10]. In the United Kingdom (UK), this annual cost to society reaches £7.4 billion, including £0.9 billion to the National Health Service [11].

To tackle this issue, national and local governments have implemented a wide range of population-level interventions aimed to improve physical activity behaviours among adults, with relative success to date [12]. However, increasing recognition has been given to the complexities of improving physical activity behaviours and that individuals are more likely to stay active if they engage in activities that can easily fit into their daily schedules (i.e., incidental physical activity) [13]. In response to this, a growing interest has emerged among policymakers on the role of local public transport and provision for active travel (that is, making journeys by physically active means) for boosting incidental physical activity, in particular cycling, and reducing adverse environmental impacts [14]. Increasing the currently relatively low rates of regular cycling [15] (i.e., cycling on most days of the week), has the potential to generate population health and wider societal and sustainable environmental benefits, such as impacts on carbon emissions, traffic congestion and air pollution [16].

In recent years, a considerable number of new cycling infrastructures have either been built or planned to be developed across Europe. For example, in Italy, the Metropolitan City of Milan has approved the ‘Cambio cycling mobility plan’ which is aimed to create 750 km of cycle paths by 2035, connecting the entire city and its surroundings [17]. In France, the budget for the ‘Paris Plan Velo’ project, which aims to make the French capital a 100% cycling city by 2026, has seen a budget increase of €250 million [18]. A similar pattern has emerged in the UK, where ambitious national and local active travel policy goals have been set [19], and more than £300 million have been allocated as part of an emergency active travel fund to build or improve existing cycling facilities with the aim of reducing pressure on public transport and supporting increase in active travel [20].

However, altering or creating new cycling infrastructure not only entails logistics and geometric feasibility-related issues [21], but typically requires large capital investment and so evaluating under what conditions these investments are economically worthwhile becomes critical. The potential returns on these investments crucially rely on the induced changes in physical activity behaviours within the local population and how these are sustained over time [22]. In a context of limited resources, identifying the minimum levels of behaviour change required for generating societal benefits that are sufficient to offset the intervention costs is important to inform future policy decisions and health-promotive actions. To address this proposition and provide context to the findings, a break-even analysis of a case study cycling network expansion project planned in Coventry (UK) was conducted.

## Methods

### Case study

A 6-km cycleway expansion has been planned in the form of the creation of a separated cycleway extending from Coventry city centre to the west side of the city [23]. Coventry is located in the West Midlands (UK) and has a population of 379,387 residents with a relatively young population; just over 65% of adults are of working age and one-third of the population are from minority ethnic groups [24]. This cycleway project is managed by the local City Council and is a regional priority route which will form part of a broader network of cycling trails in the West Midlands. Part of the route has existing cycle lanes, whereas other sections have no provision for cycling. The cost of the intervention has been estimated at £8,594,000 (2022 prices) which will be covered by a range of regional active travel and transport funds. Construction works started in mid-January 2022 and is scheduled to be completed by summer 2023 [25].

### Economic modelling

Focusing on the health-related benefits of cycling, the Health Economic Assessment Tool (HEAT, v5.0.6, November 2021) was used to address the decision problem of identifying the increase in cycling levels that would be required to achieve a benefit-cost ratio (BCR) of 1:1. The BCR is calculated as the ratio between the health-related economic benefits of the intervention resulting from changes in cycling behaviour offset by the intervention cost, that is the cost of constructing the new cycleway. The HEAT has been developed by the World Health Organization and is a quantitative tool which can estimate the monetary value of incremental benefits and risks (i.e., mortality) associated with changes in specific types of active travel-related behaviours (i.e., cycling and walking) and exposures (e.g., pollutants) in a specific geographical location and population over a defined period of time [26]. No ethical approval was required for this study as this is a model-based simulation which used aggregate-level, secondary data that were analysed anonymously.

Within the HEAT model v5.0.6, the impact of the cycling infrastructure is captured by measuring outcomes across four domains: physical activity benefits, air pollution risk, crash risk and carbon emissions, and all of these are assumed to emerge from making changes to population cycling levels and switching from motorized modes of transport. Cycling levels are measured both before and after the intervention, using five levels from ‘Daily or almost daily’ (regular cyclist) to ‘Never’. The carbon emissions are captured by using the additional costs (cost-savings) to society calculated based on damage cost estimates. These are the monetized value of the global damage caused by the incremental impact of an additional tonne of CO2 emitted [27]. The other three domains are directly captured from changes to mortality risk estimated using the value of a statistical life (VSL) approach.

VSL is an approach, often used in the transport sector, which aims to estimate the population’s willingness-to-pay for a reduction in mortality risk [28]. As required for BCR calculations using HEAT, the intervention costs (£8,594,000) were converted to international dollars ($), also referred to as purchasing power parities (PPP), using a 0.667865 coefficient [29], to $12,800,491. PPP is a well-established metric which allows for economic values expressed in different currencies to be adjusted by controlling for price level differences between countries so that they can be at par with the purchasing power of each other [30].

### Break-even economic analysis

A break-even analysis is commonly applied to determine the unit price of a product at which a company will break even [31]. This accounting method determines the point at which total costs and total revenues are equal. The break-even point is derived by dividing the fixed costs by the revenue generated (sales price per unit minus variable cost per unit). To determine the break even point in this study, this approach was adapted by defining the revenue generated as the societal benefit measured in monetary terms from increasing population cycling levels using the HEAT tool.

An iterative computational approach [32] was applied to identify the behaviour change required to offset the investment costs. Specifically, starting from a base-case behaviour change value, a sequence of improving approximate solutions were iteratively generated until a BCR of 1:1 was identified. The base-case settings and model assumptions are detailed in **S1 Appendix**.

Following the categorisation of cycling used in the HEAT tool (daily or almost daily, one to three days a week, one to three days a month, less than once a month, never), model simulations were carried out to identify the break even point for a shift from each of the bottom four categories to the top (i.e., cycling daily or almost daily). Model results were reported separately and on average, that is by averaging across the four categories.

The model simulated the intervention impact on the relevant subset of the adult population, that is among residents aged at least 18 years old who lived in proximity of the planned cycleway, who were therefore more likely to benefit from this intervention (i.e., local population subset) [33]. For the purpose of this analysis, a two-mile radius from the cycleway was considered as the intervention “catchment area” [34], which encompasses 12 of the 18 electoral wards in Coventry, with an estimated local adult population of 173,169 [35]. A 400% increase in the number of adults cycling daily or almost daily (herein referred to as ‘regular cyclists’, from 1% to 5%) was set as the base-case value of change in cycling levels for model simulation, in line with current local cycling goals [36]. This assumed that the number and length of trips remained constant. As for the remaining cycling levels, **Table 1** shows the assumed ‘pre-construction’ distribution of the local population subset, based on published statistics [37] and the classification used within the HEAT tool.

**Table 1:** ‘Pre-construction’ cycling levels in the local population subset (N = 173,169).

Cycling level	Frequency	Proportion
1	daily or almost daily	1%
2	one to three days a week	9%
3	one to three days a month	15%
4	less than once a month	25%
5	Never	50%

**Note**: The HEAT requires the user to input a percentage estimate for each of the five levels

#### Base-case model assumptions

The HEAT default and background model parameters were applied for the base-case scenario simulation [26]. Briefly, it was assumed that it would take one year from the end of construction to reach a maximum steady-state (‘take-up time’) cycling level, and that 50% of this behaviour would occur while being exposed to air pollution in moderate levels of traffic congestion. Based on default values provided by the HEAT, modal shifts from alternative means of transport were assumed to derive from the following: 50% from public transport, 30% from car and 20% from walking. International $ was used as the currency to allow for cross-country comparisons [38]. Costs and benefits were discounted at an annual 5% rate and the value of a statistical life was set at international $4,260,000 (£2,845,773). Additional model assumptions are detailed in the HEAT technical appendix [26]. All the four dimensions described above (physical activity benefits, air pollution risk, crash risk and carbon emissions) were considered over a ten-year time horizon to align with current active travel targets by the regional authority for transport [35].

#### Sensitivity analysis

By default, the HEAT applies several values and assumptions that are based on the best available evidence and expert consensus [26]. However, this tool also allows the users to override default values and assumptions, although some only within predefined ranges / categories. In line with previous research using the HEAT [39] and following method guidance on health economic assessments of population health interventions to inform decision-making [40], the base-case results were tested for sensitivity to variations to: age of the target population (20–44 and 45–64 years old cohorts), VSL (+/-20%), discount rate (+/- 1.50%), time horizon (5 and 15 years), as well as considered cycling outcome measures (i.e., +/-20% trip length and number of trips), take-up time (two, three and five years) and modal shift-related assumptions (i.e., proportion of people shifting from other means of transport and proportion cycling ‘in traffic’). For each variation, both the respective new BCR and additional change in cycling behaviour–from the level 2 to the first cycling category–required to reach a break-even were estimated.

## Results

### Break even analysis

The estimated change in cycling levels required to break even on the intervention cost ($12,800,491) under base-case assumptions are shown in **Table 2**. This table presents four scenarios, each depicting the number and proportion of adults required to change behaviour from different baseline cycling levels. For example, in scenario 1, the model depicts how many adults are required to break even when the shift is focused on adults moving from Level 2 –cycling one to three days a week (at baseline) to Level 1 –cycling daily or almost daily (over ten years).

**Table 2:** Break even point estimates of increase in regular cyclists (level 1).

**N = 173,169**	**Break even point**		**Economic impact**			
	**Post-intervention cycling levels**		**Additional value**	**deaths**	**additional value**	**Domain**
**Scenario 1 -shift from level 2**	daily or almost daily	2.242%		-5.00	$ 15,743,658	Physical activity benefits
	one to three days a week	7.758%		0.26	$ -801,133	Air pollution
	one to three days a month	15%		0.68	$ -2,212,084	Crash risk
	less than once a month	25%		tons of CO2	**additional value**	Carbon emissions
	never	50%		-721	$ 70,049	
	Required behaviour change	New regular cyclists				
	1.242%	**2,151**	** **			
	**Post-intervention cycling levels**		**Additional value**	**deaths**	**additional value**	**Domain**
**Scenario 2—Shift from level 3**	daily or almost daily	1.863%		-5.00	$ 15,763,533	Physical activity benefits
	one to three days a week	9%		0.25	$ -803,237	Air pollution
	one to three days a month	14.137%		0.67	$ -2,229,988	Crash risk
	less than once a month	25%		tons of CO2	**additional value**	Carbon emissions
	never	50%		-717	$ 70,183	
	Required behaviour change	New regular cyclists				
	0.863%	**1,494**				
	**Post-intervention cycling levels**		**Additional value**	**deaths**	**additional value**	**Domain**
**Scenario 3—Shift from level 4**	daily or almost daily	1.808%		-5.00	$ 15,764,522	Physical activity benefits
	one to three days a week	9%		0.25	$ -805,296	Air pollution
	one to three days a month	15%		0.67	$ -2,229,124	Crash risk
	less than once a month	24.192%		tons of CO2	**additional value**	Carbon emissions
	never	50%		-719	$ 70,388	
	Required behaviour change	New regular cyclists				
	0.808%	**1,399**				
	**Post-intervention cycling levels**		**Additional value**	**deaths**	**additional value**	**Domain**
**Scenario 4—Shift from level 5**	daily or almost daily	1.793%		-5.00	$ 15,764,522	Physical activity benefits
	one to three days a week	9%		0.25	$ -805,296	Air pollution
	one to three days a month	15%		0.67	$ -2,229,124	Crash risk
	less than once a month	25%		tons of CO2	**additional value**	Carbon emissions
	never	49.207%		-719	$ 70,388	
	Required behaviour change	New regular cyclists				
	0.793%	**1,373**				

On average, an additional 1,604 (that is, 0.924% of 173,649) regular cyclists (cycling every day or almost daily) were found to be required to reach a BCR of 1:1. That is 267 cyclists per km of cycleway constructed. In monetary value, therefore, this corresponds to an annual $798 (£ 533) generated per additional regular cyclist over a ten-year time horizon.

**Table 2** shows that, depending on the baseline cycling level, ranging from Level 5 (never cycle) to Level 2 (cycle one to three days a week), the number of regular cyclists required to break even ranges across scenarios from 1,373 (0.793%) to 2,151 (1.242%). These results show the decreasing marginal benefit from improving cycling among those who never cycled compared to those who already occasionally cycled, where a 56% difference in the number of new regular cyclists emerges. **Table 2** also shows that the benefits induced by increased physical activity levels contributed the most to the total economic gain and that these benefits were attenuated by negative effects from increased crash risk and exposure to air pollution.

The relative contribution of the four domains changed only marginally across the shifting of cycling categories, with physical activity benefits contributing more and air pollution contributing less, in relative terms, as the model moved towards the ‘never’ cycling category and with this difference being undetectable between the fourth and fifth baseline cycling category. Moreover, **Table 2** shows that the additional economic value generated by lower carbon emissions represented only a fraction (~0.5%) of the total economic impact.

### Sensitivity analysis

**Table 3** shows that the base-case estimates were sensitive to variations to the age group and time horizon of the evaluation, as indicated by the respective BCRs deviating the most from the equilibrium value of 1.00. Compared to the base-case, if the change in behaviour was only within adults aged between 20 and 44 years, an extra 11,949 adults would be needed to break even, instead of the 2,151 adults within the base-case. That is 5.6 times as many adults would be required to transition from cycling between level 2 (one and three days a week), to level 1 (at least four days a week). Furthermore, if the time horizon is shortened from 10 to 5 years, then 3,183 more regular cyclists (Level 1) would be required to break even.

**Table 3:** Sensitivity analysis.

	Model parameters and assumptions	Variation	BCR	Population % of regular cyclists	Absolute number of regular cyclists	additional n. of regular cyclists relative to base-case
	Age group	20–44 years	0.173	7.900%	13,680	9,798
		45–64 years	1.976	1.615%	2,797	-1,086
	Value of Statistical Life	$ 3,408,800	0.805	2.550%	4,416	533
		$ 5,113,200	1.203	2.035%	3,524	-358
	Discount rate	6.50%	0.914	2.350%	4,069	187
		3.50%	1.094	2.130%	3,688	-194
	Time horizon	5 years	0.403	4.080%	7,065	3,183
		15 years	1.469	1.845%	3,195	-687
**Cycling outcomes**	Baseline trip length per day	3.28 km	0.797	2.550%	4,416	533
		4.92 km	1.203	2.035%	3,524	-358
	Baseline number of trips per day	1.6 trips	0.797	2.550%	4,416	533
		2.4 trips	1.203	2.035%	3,524	-358
	Post trip length	3.28 km	0.722	4.910%	8,503	4,620
		4.92 km	2.726	0.458%	793	-3,089
	Post number of trips per day	1.6 trips	0.722	4.910%	8,503	4,620
		2.4 trips	2.726	0.458%	793	-3,089
	Take-up time for active travel demand	2 years	0.922	2.340%	4,052	170
		3 years	0.851	2.455%	4,251	369
		5 years	0.713	2.745%	4,753	871
	Proportion “in traffic”	100.00%	0.977	2.280%	3,948	66

**Note**: Base-case number of extra cyclists required to reach a 1:1 BCR = 2,151. BCR = benefit-cost ratio. y = years.

A 20% change to either the VSL, the baseline trip length or number of trips corresponded to a somewhat comparable change to the number of adults required to change behaviour. If, however, the 1,604 increment in regular cyclists was to be combined with a 20% reduction either in the average trip length or number of trips, then that would not suffice to offset the intervention cost (BCR = 0.722). In that case, 4,620 extra regular cyclists would be required. Conversely, if either the average trip length or number of trips increased by 20%, then 938 fewer regular cyclists (that is a shift from 1% to 0.458% level 1) would be required to break even.

**Table 3** also shows how the BCR decreases as the assumed take-up time for active travel is more conservative, from 0.922 at two years to 0.713 at five years, an additional 871 regular cyclists will be required to offset the cost of the investment over a ten-year time horizon. Due to the low marginal contribution from reducing carbon emissions on the total economic impact, any assumptions linked to the modal shifts had either a limited or no impact (proportion of people shifting from other means of transport) on the base-case results.

## Discussion

The aim of this paper was to estimate the value generated from each additional regular cyclist to enable an estimation of the population behaviour change requirement to justify investing in cycling infrastructure. To this end, a break-even analysis was conducted of a planned cycling infrastructure project taking place in Coventry (UK), and the number and proportion of new regular cyclists needed for the intervention cost to be offset by the economic gains generated.

The model estimated that each additional regular cyclist generated, on average, international $798 (£533), per year, over a ten-year time horizon. Therefore, to achieve a benefit-cost ratio of 1:1 on the construction of a 6 km-long separated cycleway, an additional 1,604 regular cyclists, that is 267 cyclists per km of cycleway constructed, would be required. This will be 20% lower or higher depending on baseline cycling levels. These estimates, however, are particularly sensitive to variations to assumptions made such as the age group, cycling volume (trip length and number of trips) and evaluation time horizon.

### Comparison with previous studies

To the best of our knowledge, this is the first break-even economic analysis of a cycling infrastructure investment and more broadly a physical activity promotion intervention [41]. An example of such type of assessment can be found within the obesity literature, where Bates et al. (2022) estimated the maximum justifiable cost for a weight loss maintenance intervention for individuals at different BMI and type II diabetes risk [42]. Similarly, our model estimated the expected economic value of an additional regular cyclist and the proportion and number of cyclists required to achieve a BCR of 1:1 depending on four baseline cycling levels. A growing literature on the economic implications of cycling interventions and policies, predominantly cycling infrastructure, has rapidly accumulated over the last two decades, with the evaluation scope shifting from transport-related outcomes (e.g., traffic congestion and crash risk) [43] to include health effects, namely, physical activity benefits and exposure to air pollution and carbon emissions [44, 45]. Overall, most of the evaluated planned or implemented cycling infrastructure interventions have been found to provide good value for money, either in the form of BCRs greater than one or incremental cost-effectiveness ratios below the given willingness to pay threshold for an additional quality- or disability-adjusted life year.

As an example of an economic analysis from the UK using the HEAT tool, Cope et al. (2010) assessed the return on investment of the English Cycling Demonstration Town investment programme [46]. This programme consisted of a combination of physical infrastructure, promotion and other smart measures and, with an estimated cost of £18 million over three years (2005–2007), was found to generate a 2.6 to 3.5:1 BCR. Another evaluation where the HEAT tool was applied was that by Deenihan and Caulfield (2014), who examined the health and economic benefits from the construction of a new separated cycleway in Ireland [47]. This cycle route was planned along a disused towpath of a canal approximately 60 km long and fully separated from any vehicular traffic, with an estimated €12,000,000 in construction costs. The authors estimated that the investment would lead to benefit–cost ratios ranging between 2.22:1 and 11.77:1. A recent study by Whitehurst et al (2021) used the HEAT to assess the health-related economic impact of bicycle infrastructure investments in three Canadian cities (Victoria, Kelowna and Halifax). Using scenario analysis, these authors estimated the BCRs to be between 1.7:1 (Victoria) and 2.1:1 (Halifax) under a 2%, and 3.9:1 (Victoria) and 4.9:1 (Halifax), under a 5% increase in bicycling mode share scenarios.

Two recent economic analyses from Sweden and Norway focused the economic evaluation on the healthcare cost implications and long-term impacts on morbidity and mortality quantified in disability- and quality-adjusted life years, respectively. Kriit et al. (2019) assessed the cost-effectiveness of a €101 million investment in urban cycling infrastructure proposed by the local municipality in Stockholm who aimed to achieve a 15% increase in the number of cycling commuters by 2030 [48]. For this analysis, the authors used a comparative risk assessment approach whereby the number of attributable disability-adjusted life years and long-term healthcare costs (added and averted) due to a change in physical activity, air pollution and traffic accidents from an estimated number of additional cyclists were computed and found that the intervention was cost-effective from a healthcare sector perspective. Comparably, Lamu at al. (2021) assessed the cost-effectiveness of expanding the current cycle network in Oslo by 100km at $3.5 million per km and an annual maintenance cost of 7% by developing a state-transition Markov model to simulate the impact of changes in cycling, and therefore overall physical activity levels, on four related diseases (cancer, coronary heart disease, stroke and type II diabetes) and associated costs and quality-adjusted life years [49]. Compared to no intervention, the 100km cycling expansion program was deemed cost-effective, at an incremental $16,575 per quality-adjusted life year gained.

There are methodological variations between these economic studies that used the HEAT in terms of modelling methods and assumptions which limits the comparability of study findings. With this study, an alternative way of using the HEAT model has been presented that offers an ex-ante analysis to estimate the number of additional cyclists required to break even when some of the expected health-related economic benefits of the cycling infrastructure investment are considered. This provides useful policy information so that decision makers know in advance what level of behaviour change is required to justify investment in cycling infrastructure.

### Strengths and limitations

However, while the sensitivity of break-even results was explored, in practice, constructing a new cycleway will likely attract change in behaviour from a combination of the four baseline levels. In addition, and in common with previous HEAT-based modelling studies [50], the model lacked a probabilistic sensitivity analysis which would have quantified the level of confidence in the outputs in relation to the combined uncertainty in the model inputs. This was not possible as the HEAT tool, in its current form, does not include this function or an option for model adaptation.

As a model-based assessment, the presented estimates are a function of the structural parameters and assumptions underlying the economic model employed. The HEAT is built using a comparative risk assessment approach, based on aggregate, population-level data, where a direct comparison between two steady states (i.e., base-case vs the intervention) is made without formally modelling recurrent events or interactions between individuals and their environment [51]. Model accuracy may be therefore hindered. Nonetheless, HEAT provides an important tool to conduct early economic modelling and break even analyses of cycling interventions [52], such as that reported in this study. As acknowledged by its authors, this tool aims to provide rough order-of-magnitude estimates that are sufficiently accurate and precise enough to support the necessary political discourse around the value of investments in active travel [26].

The model presented in this paper only considered mortality impacts mediated by changes to physical activity, crash risk and air pollution exposure and only cycling behaviours. Therefore, the estimates are likely to be conservative as they do not consider health and wellbeing morbidity effects. When deciding which tool to use to conduct the analysis, among different criteria including relevance, model accuracy and flexibility, primary importance was given to transparency and reproducibility of findings which are crucial to strengthen evidence and build upon existing work [53]. In fact, other open-access health assessment tools focusing on cycling behaviour exist. However, in their current versions, these tools either consider a mere health perspective (i.e., chronic disease reduction risk) [54], or do not formally account for the investment costs, hence limiting the ability of users to derive BCR ratios [55]. Furthermore, as well as being regularly updated over the years, the HEAT has been widely applied across a large number of countries and been endorsed by government agencies around the world [50]. This tool is designed for non-expert users with little expertise in the active travel field, such as policymakers whose informing processes and decision-making practices this study aims to influence.

The generalisability of the results to different decision-making contexts depends upon a number of modelling assumptions and key contextual factors. Regarding the approximate estimate of the number of new regular cyclists required to break even on the construction of a new separated cycleway, the 1,604 value (267 per km) was calculated based on intervention costs measured in purchasing power parity (PPP) terms. Nevertheless, PPP is only a proxy adjustment factor and differences in consumption and production patterns between countries make the identification of a common ‘standard’ basket of goods difficult [56]. Moreover, unit costs per km constructed are unlikely to be homogeneous between settings, as they tend to be largely dependent on the specific site (e.g., along a canal, across a park or alongside a road) and location (e.g., rural or urban) in which the cycling infrastructure is constructed.

Contextual factors will also play a key moderating role on the benefits. Different traffic conditions, and consequent air pollution and crash risk to which cyclists are exposed to when using the new infrastructure will impact the economic gains and therefore the additional value per new cyclist. In this respect, the HEAT provides a flexible tool to allow analysts to specify both the geographical scale (e.g., national, city or sub-city level) and location from countries and regions around the world, and therefore conduct health impact assessments using local-level data. Furthermore, the tool includes location-specific air pollution exposure and crash risks derived from literature reviews and evidence syntheses, hence allowing for accurate economic estimations of these effects.

The extent of economic gain depends on the value assigned to the modelled end point, that is mortality. At the time of writing, the HEAT did not account for other relevant health effects such as morbidity, although plans are in place to address this limitation [26]. By focusing only on some of the health-related economic benefits, this study took a relative narrow perspective in that it did not include other economically relevant non-health effects, such as time savings benefits and disbenefits which have the potential to significantly affect the presented estimates [57]. Hence, careful consideration needs to be given when using these results as input for future business cases and other applications. In addition, this study is also narrow in scope–e.g., by only considering the health impacts on adults–and only complements the more complex and comprehensive transport appraisal methodology [58] which is typically used by policy-makers, including local authorities, to support active travel investment decisions.

Other economic modelling tools comparable to the HEAT have been developed. These include the Impacts of Cycling Tool (ICT) [59], which is an open source model with a web interface for visualising travel patterns in England and compares the impact of different cycling uptake levels at the population level and across subgroups. The ICT however does not formally consider the increased exposure to air pollution and crash risks from increased cycling, nor it allows for cost-benefit assessments to be conducted. Another available tool is the Integrated Transport and Health Impact Modelling Tool (ITHIM) [54] which enables integrated assessments of the health effects of transport scenarios and policies at the urban and national level. While comparable in scope to the HEAT, within the ITHIM, the health effects are presented both as number of attributable deaths (similar to the HEAT) and disability-adjusted life-years, with background burden data for study areas being estimated from Global Burden of Disease studies [60]. Several international research and policy collaborations involving the ITHIM are underway and future plans include making its model and its multiple versions open source [54].

Transport studies typically use a VSL approach derived using willingness to pay methods. However, other valuation approaches typify public health decision-making contexts where extra-welfarism dominates [61]. Indeed, in line with previous work [62, 63] and transcending traditional welfarism, this study focused on health as a merit good. Furthermore, in the UK and increasingly in many countries around the world, recognition has been given to the importance and social value of reducing existing unfair and unjust health inequalities which could not be incorporated into this analysis. While a growing body of empirical literature has been generated [64], a recent example is the study by Lamu et al. (2021), for which the researchers developed a decision-analytic model to assess the cost-effectiveness of the intervention by socio-economic status and under what circumstances the intervention would reduce baseline inequality and increase social welfare.

Despite these contributions to the cost-effectiveness literature on cycling interventions, it is important to note that, in practice, at the local level active travel investment decisions are not often based on such evidence [65]. This may be due to the large number of contextual factors that can affect the decision, in particular the type of project being evaluated. Against this backdrop, the break-even analysis results presented here could be used to inform the planning and budgeting of future cycling infrastructure investments.

## Conclusions

Policymakers should consider using these approximate order-of-magnitude, yet reproducible estimates to set corresponding population cycling targets when planning the creation or enhancement of cycling infrastructure. This will ensure that the investment is justifiable from an economic sustainability perspective on cost-neutrality grounds. Future research should consider using the economic modelling approach illustrated in this study to build a relevant evidence base for decision-makers and replicating the analysis for other intervention contexts and healthy behaviours.

## Supporting information

S1 Appendix Default and background input parameters and assumptions applied for base-case analysis. (DOCX)
