# Supplementary material for: How much behaviour change is required for the investment in cycling infrastructure to be sustainable? A break-even analysis
Source: PLoS One. 2023 Apr 19;18(4):e0284634. doi: 10.1371/journal.pone.0284634 (PMC10115289; doi:10.1371/journal.pone.0284634)
Supplement: S1 Appendix — (DOCX) [file pone.0284634.s001.docx]

| **HEAT tool v5.0.6 (November 2021) window** | **Input / choice** |
| --- | --- |
| ***User interface options*** | |
| User experience | Full user interface |
| ***Active travel modes*** | |
| Active travel modes | Cycling |
| ***Geographical scale*** | |
| Country | The United Kingdom |
| Geographical level | City |
| Location | Coventry |
| Assessment located at the sub-city level | Yes |
| ***Comparison and time scale*** | |
| Type of assessment | Two cases |
| Year of reference case | 2022 |
| Year for comparison case | 2032 |
| Time horizon | 10 years |
| ***Impacts*** | |
| Impacts considered in the assessment | physical activity, air pollution, crash risk and carbon emissions |
| ***Motorized modes*** | |
| Motorized travel modes | no data |
| Traffic conditions | some congestion |
| ***Study specific input parameters*** | |
| Population | General population |
| Age range | 20-64 years |
| Data source | Population survey |
| Data type | Frequency category |
| Number of trips per day | 2 |
| Trip length | 4.1 km |
| Cycling data for the reference case (%) | |
| Daily or almost daily | 1 |
| 1-3 days per week | 9 |
| 1-3 days per month | 15 |
| Less than once per month | 25 |
| Never | 50 |
| Cycling data for the comparison case (%) | |
| Daily or almost daily | 5 |
| 1-3 days per week | 8 |
| 1-3 days per month | 14 |
| Less than once per month | 24 |
| Never | 49 |
| Population size (number of individuals) | 173,649 |
| ***General adjustments*** | |
| Proportion excluded (exclude walking or cycling due to factors unrelated to the assessed intervention) | 0% |
| Temporal & spatial adjustment (to adjust data as necessary to reflect long-term averages) | 0% |
| Take-up time for active travel demand (which year of your assessment the "comparison level" of active travel is reached) | 1 year |
| Proportion of new trips | 0% |
| Proportion of reassigned trips | 0% |
| Proportion for transport | 50% |
| Proportion shifted from driving | 30% |
| Proportion shifted from public transport | 50% |
| Proportion shifted from walking | 20% |
| Proportion “in traffic” | 50% |
| Substitution of physical activity | 0% |
| ***Additional parameters*** | |
| All-cause mortality rate for cycling | 235 (per 100,000 population) |
| PM2.5 concentration used in HEAT assessment (ug/m3) | 10 |
| Road fatalities for cycling | 111.6 (per 100,000 population) |
| Annual total of cycling for the population | 5,220,666,292 (distance in km) |
| Fatality rate | 2.1 (per 100,000 population) |
| Value of Statistical Life (international $) | 4,260,000 |
| Investment cost (international $) | 12,800,491 |
| Discount year and rate | 2022, 5% |
| ***Default values*** | |
| Carbon value (2023, USD2014/tCO2e) | 110 |
| Carbon value (2032, USD2014/tCO2e) | 145 |
| Average occupancy rate for cars | 2 persons |
| Proportion shifted from car to bike | 50% |
| Proportion shifted from public transport to bike | 30% |
| Proportion shifted from walk to bike | 20% |
| Average cycling speed | 14 km/h |
| ***Background values*** | |
| Time need to obtain full health in crash module (years) | 0 |
| Time need to obtain full health in two cases assessment (years) | 5 |
| Relative risk for mortality and bike | 0.90 |
| Relative risk of air pollution (PM 2.5) and mortality | 1.08 |
